# Protective effect of epigallocatechin gallate, a major constituent of green tea, against renal ischemia–reperfusion injury in rats

**DOI:** 10.1007/s11255-015-1030-0

**Published:** 2015-06-28

**Authors:** Jun Lv, Min Feng, LiLi Zhang, Xia Wan, Yu Chun Zeng, Pei Fen Liang, An Ping Xu

**Affiliations:** Department of Nephrology, Sun Yat-Sen Memorial Hospital, Sun Yat-Sen University, Guangzhou, People’s Republic of China

**Keywords:** EGCG, Inflammation, Apoptosis, Renal ischemia–reperfusion injury

## Abstract

**Background:**

Renal ischemia–reperfusion (I/R) injury plays an important role in the acute kidney injury. The pathogenetic mechanisms potential I/R injury is involved in apoptosis and inflammation. Epigallocatechin gallate (EGCG), a major constituent of green tea, has been shown to have anti-inflammatory and anti-apoptotic activities. This study aimed to explore the underlying effects and mechanisms of EGCG on renal I/R injury in a rat model.

**Materials and methods:**

We induced renal I/R injury in SD rats by clamping the left renal artery for 45 min followed by 24-h reperfusion, along with a contralateral nephrectomy. We randomly allocated 30 rats to three groups (*n* = 10): sham group, IRI group, and EGCG group. We preconditioned rats intraperitoneally with EGCG (50 mg/kg) or vehicle (50 mg/kg) 45 min before inducing renal ischemia. We collected serum and kidneys at 24 h after reperfusion. Renal function and histologic damage were assessed. We also determined markers of inflammation and apoptosis in kidneys or serum.

**Results:**

EGCG pretreatment can significantly reduce renal dysfunction, histologic change and the expression of tumor necrosis factor-α, IL-1β, IL-6, Bax and cleavage caspase 3 induced by I/R injury and increase the expression of Bax and caspase 3. Moreover, EGCG pretreatment can further induce the activation of p38 mitogen-activated protein kinase in kidney, with no influence on the expression of p38.

**Conclusions:**

EGCG treatment can decrease renal ischemia–reperfusion injury by suppressing inflammation and cell apoptosis. Thus, EGCG may represent a potential strategy to reduce renal I/R injury.

## Introduction

Renal ischemia–reperfusion (I/R) injury, a main cause of acute renal failure, leads to unbelievably high morbidity and mortality and is common in those with trauma, shock, transplantation, renal resection, and so on [1]. Research shows that pathogenesis of renal I/R injury is involved in inflammation, cell apoptosis, necrosis, reactive oxygen species, and so on [2]. But the precise mechanism is still unclear. Obviously, it is reported that inflammation and cell apoptosis play a vital role in renal I/R injury [3]. Therefore, suppressing inflammation and apoptosis may be an effective approach to alleviate renal I/R injury.

Mitogen-activated protein kinase (MAPK) is a traditional signal pathway that plays an important role in regulating NF-κB activation in renal ischemia–reperfusion injury [4]. Moreover, NF-κB activation can induce inflammation and cell apoptosis [5]. Therefore, regulating MAPK signaling pathway may be a promising intervention to decrease renal I/R injury.

**Fig. 1:**
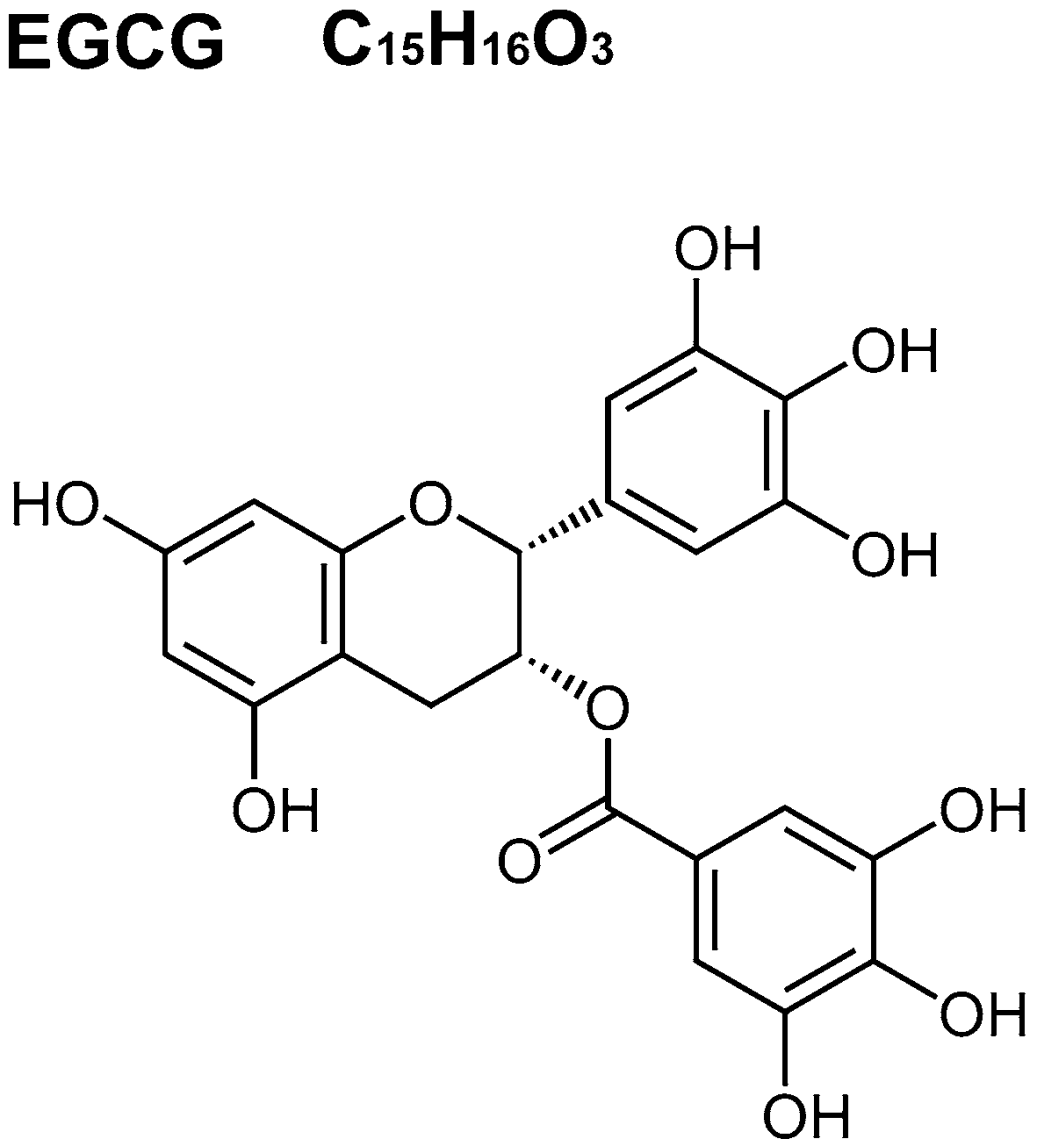
Structure of EGCG

Epigallocatechin gallate (EGCG, C_23_H_20_O_11_, Fig. 1), a major constituent of green tea, is extracted from Camellia sinensis plant [6]. EGCG has been shown to have anti-tumor, anti-inflammatory and anti-apoptosis properties in multiple cells [6]. Because of these bioactivities, it is speculated that EGCG may exert therapeutic benefit in many apoptotic and inflammatory diseases such as ischemia–reperfusion injury, atherosclerosis and transplantation [7]. Therefore, the aim of the study was to explore the effects and mechanisms of EGCG on renal I/R injury.

## Subjects and methods

### Animals and experimental groups

Male Sprague–Dawley rats (200–250 g) were purchased from Hua Fukang Experimental Animal Center, Beijing, China, and maintained under standard conditions until the experiments were performed. Animals were fed under standard diet and water ad libitum. All procedures were performed in accordance with the principles of the Guidelines of Animal Experimentation at Zhong Shan University. EGCG was purchased from Sigma, its purity is over 98 %, and it is dissolved in saline.

The rats were randomly divided into three groups (*n* = 10 per group): (1) sham group: all the surgical steps were performed; however, renal I/R was not induced. The animals were kept under anesthesia for the duration of the renal I/R procedure. (2) IRI group: renal I/R was induced and this group was pretreated with saline (50 mg/kg, ip). (3) EGCG group: renal I/R was induced and this group was pretreated with EGCG (50 mg/kg). EGCG and saline were pretreated 45 min before inducing renal ischemia by intraperitoneal injections.

### Renal I/R injury model

Rats were first anesthetized by an intraperitoneal injection of 1 % sodium pentobarbital (40 mg/kg). Renal I/R injury was induced as previously depicted [8]. A midline laparotomy incision was made, followed by a right nephrectomy. The isolated left renal artery was blocked with a non-traumatic microvascular clamp for 45 min followed by 24-h reperfusion in a controlled-environment room with food and water freely available. Sham-operated rats underwent laparotomy without occluding renal artery as I/R and EGCG groups. The rats were killed 24 h after reperfusion, and their kidneys and serum were harvested for further study.

### Assessment of renal function

Blood samples were obtained from the inferior vena cava 24 h after reperfusion. Blood urea nitrogen (BUN) and serum creatinine (Cr) levels were assayed in the core laboratory of SUN Yi-Xian Hospital for assessing renal function.

### Histologic analysis

Kidney tissue samples were fixed in formalin and then embedded in paraffin, and renal sections were next prepared and subjected to hematoxylin–eosin (HE) staining as reported [9]. The histopathologic changes in the cortex and medulla were evaluated by a pathologist in a blinded fashion using a five-point quantitative scale according to the degree of tubular necrosis, hemorrhage and cast formation as follows: 0, <10 %; 1, 10–25 %; 2, 25–50 %; 3, 50–75 %; and 4, 75–100 % [9].

### Immunoblot analysis

p-p38 (CST, USA, 1:500), p38 (CST, USA, 1:1000), p-p65 (CST, USA, 1:500), p65 (CST, USA, 1:1000), Bax (CST, USA, 1:500), BCL-2 (CST, USA, 1:1000), caspase 3 (CST, USA, 1:1000) and cleavage caspase 3 (CST, USA, 1:1000) were used to probe the membranes, followed by incubation with an HRP-conjugated secondary antibody (West Grove, PA). β-Actin (Abmart, China, 1:1000) was used for normalization. The reactive bands were visualized using the ECL-Plus Reagent (Amersham, Piscataway, NJ) as instructed. The density of each band was quantified using the Labworks image acquisition platform and its related analytic software (UVP, USA). Expression in kidney tissues was detected by immunoblot analysis as previously depicted [4].

### Real-time PCR analysis

Total RNA was isolated from renal tissues using Trizol according to the manufacturer’s instructions (Takara, Japan). Four micrograms of total RNA was reverse-transcribed into cDNA using the PrimeScript RT Master Mix (Takara, Japan) as instructed. Real-time PCR amplifications were carried out using the ABI 7500 system (Applied Biosystems, USA) for rat TNF-α, IL-1β and IL-6. The following pairs of primers were used for PCR amplification: TNF-α forward primer, 5 0 -CCTGTAGCCCACGTCGTAGCAAA-3, TNF-α reverse primer, 5-AGCGCTGAGTTGGTCCCCCT-3; NM 012675.3 1687 bp; IL-1β forward primer, 5-AGCTGGAGAGTGTGGATCCCAAGC-3, IL-1β reverse primer, 5–AGCGACCTGTCTTGGCCGAGG-3, NM 031512.20,1339 bp; IL-6 forward primer, 5-CTGCAAGAGACTTCCATCCAG-3, IL-6 reverse primer, 5-AGCGCTGAGTTGGTCCCCCT-3; NM 012675.3 1687 bp; IL-6 forward primer, 5-CCTGTAGCCCACGTCGTAGCAAA-3, IL-6 reverse primer, 5-AGTGGTATAGACAGGTCTGTTGG-3, NM 012589.2, 1045 bp; β-actin forward primer, 5-AGAGGGAAATCGTGCGTGAC-3, β-actin reverse primer, 5-CAATAGTGATGACCTGGCCGT-3, NM 031144.3, 1293 bp. PCR was conducted at 95 °C for 30 s, followed by 40 cycles at 95 °C for 5 s, 60 °C for 34 s and 95 °C for 15 s. The amount of mRNA for each gene was normalized by β-actin, and the relative expression levels were calculated using the 2^−ΔΔCt^ method as reported [10].

### ELISA analysis

Levels of the inflammatory mediators (TNF-α, IL-6 and IL-1β) in the serum were quantified using specific ELISA kits for mice according to the manufacturer’s instructions (Biosource International Inc, USA).

### Statistical analysis

Data are expressed as mean ± SD. Statistical analysis was carried out with unpaired *t* test or analysis of variance (ANOVA) for multiple comparisons. In all cases, *P* value <0.05 was considered statistically significant.

## Results

### EGCG pretreatment significantly alleviates I/R-induced renal dysfunction

**Fig. 2:**
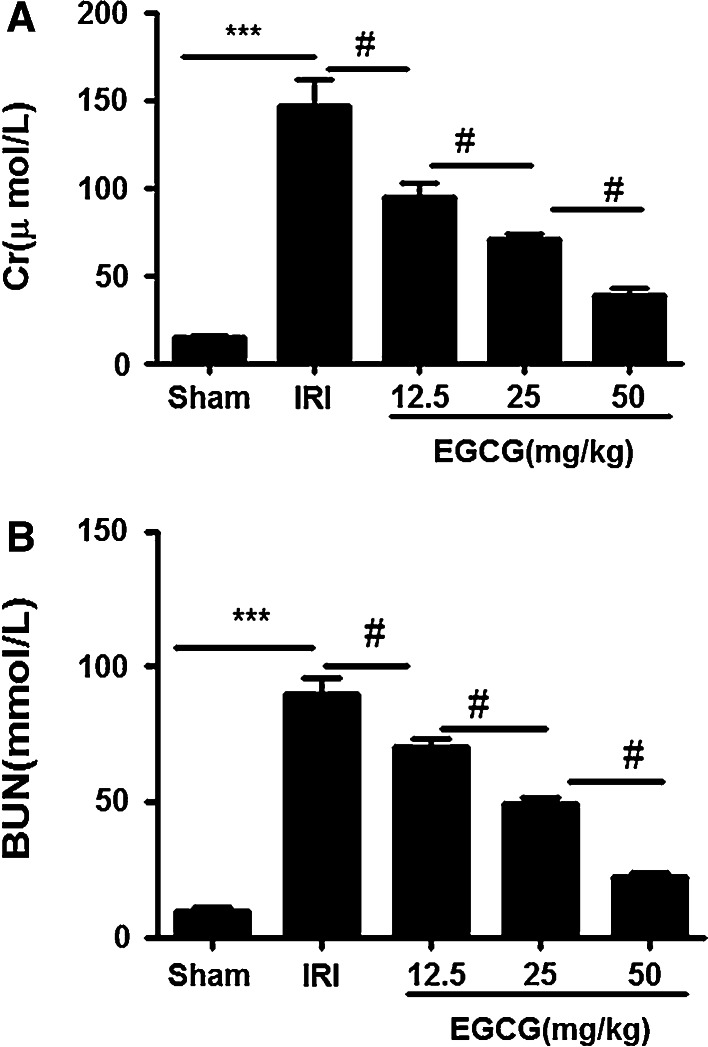
Effects of EGCG pretreatment on alterations of renal function following renal I/R-induced injury. Serum creatinine (**a**) and plasma urea (**b**) were measured to assess the renoprotective effect of against renal I/R EGCG pretreatment injury in the sham, IRI and EGCG groups. Data were represented as mean ± SEM (*n* = 10). ***P < 0.001 (IRI vs. sham); ^#^ *P* < 0.05 (IRI vs. EGCG)

We assessed Cr and BUN levels in the serum 24 h after reperfusion. Compared with the rats in sham group, the rats in the IRI group showed high level of in Cr and BUN (*P* < 0.001). However, EGCG pretreatment significantly reduced both Cr and BUN levels 24 h after I/R injury in a dose-dependent manner; the best concentration of EGCG for renoprotective effect is 50 mg/kg (*P* < 0.05) (Fig. 2a, b).

### EGCG pretreatment significantly reduces I/R-induced histologic damage in the IRI

**Fig. 3:**
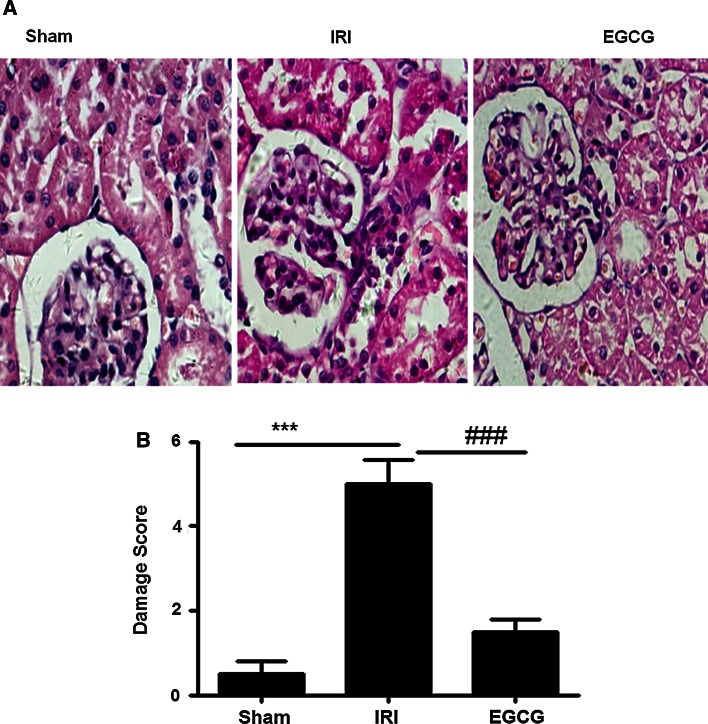
Effects of EGCG pretreatment on I/R-induced renal histologic change. Representative microphotographs were taken from the kidneys of the sham, IRI and EGCG groups at the time point of 24 h after renal I/R. Histopathologic examination was performed using HE staining (**a**). Semiquantitative assessment of the histologic lesions based on tubular necrosis (**b**). Data were represented as mean ± SEM (*n* = 10). ***P < 0.001 (IRI vs. sham); ^###^ *P* < 0.001 (IRI vs. EGCG)

To ensure the renoprotective effect of EGCG, we examined the renal histologic changes 24 h after reperfusion. Kidneys from sham group showed nearly normal tubular histology. However, kidneys from I/R rats showed significant renal histologic damage as demonstrated by widespread degeneration tubular architecture, tubular cell swelling, tubular dilation, tubular necrosis and inflammatory cell infiltration. In contrast, EGCG treatment significantly reduced histologic damage 24 h after reperfusion as demonstrated by less degeneration tubular architecture, tubular cell swelling, tubular dilation, tubular necrosis and inflammatory cell infiltration (Fig. 3a). Figure 3b shows the semiquantitative histopathologic scores of all three groups. EGCG pretreatment significantly attenuated the I/R-induced increase in histopathologic scores (*P* < 0.001).

### EGCG treatment ameliorates I/R-induced inflammation

**Fig. 4:**
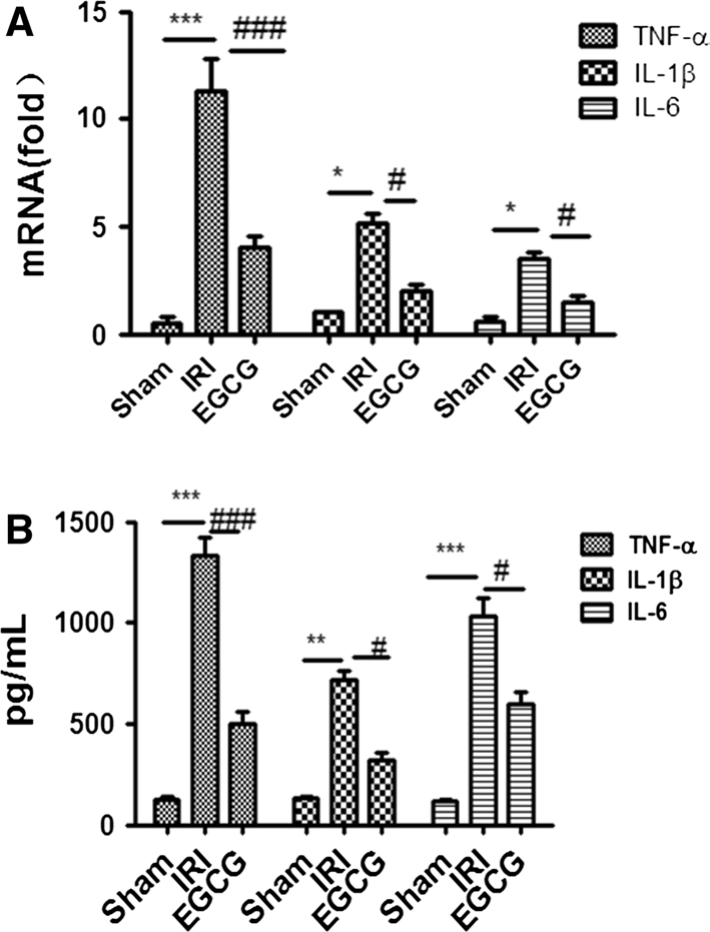
Effects of EGCG pretreatment on the expression of pro-inflammatory cytokine after renal ischemia–reperfusion injury (IRI). RT-PCR was employed to assess the expression of pro-inflammatory cytokine tumor necrosis factor-α (TNF-α), interleukin-6 (IL-6) and IL-1β in the kidney after IRI. Data were represented as mean ± SEM (*n* = 10). ***P < 0.001, *P < 0.05 (IRI vs. sham); ^###^ *P* < 0.001, ^#^ *P* < 0.05 (IRI vs. EGCG)

To explore the mechanism of EGCG decreasing renal dysfunction, we assessed the expression of pro-inflammatory cytokines TNF-α, IL-6 and IL-1β in the serum and renal tissues in renal I/R injury. The serum and kidneys from I/R rats displayed higher expression level of TNF-α, IL-6 and IL-1β than those from sham group (*P* < 0.05). However, EGCG treatment can significantly suppress the increase in pro-inflammatory cytokines in the serum and renal tissue, as shown by lower levels of TNF-α, IL-6 and IL-1β in EGCG group than in IRI group (*P* < 0.05, Fig. 4). These results implied that EGCG pretreatment could suppress I/R-induced inflammation.

### EGCG treatment ameliorates I/R-induced apoptosis

**Fig. 5:**
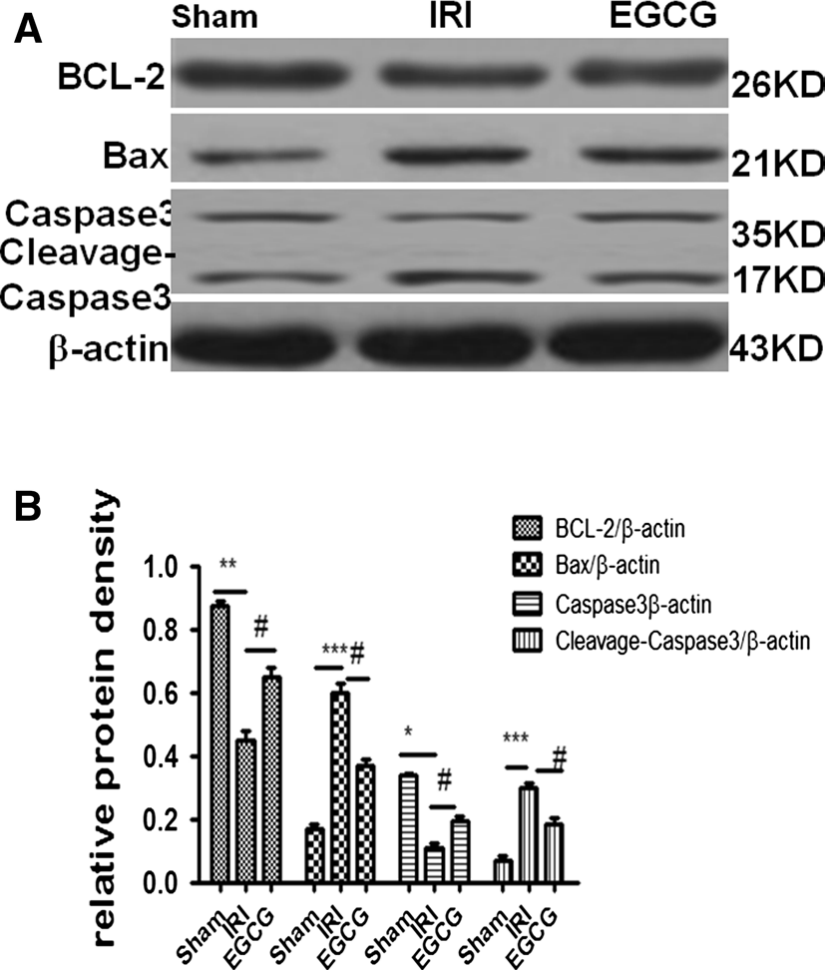
Effects of EGCG pretreatment on the expression of Bax, BCL-2, caspase 3 and cleavage caspase 3 following renal I/R-induced injury. Western blot analysis was employed to assess the expression Bax, BCL-2, caspase 3 and cleavage caspase 3. **a** A representative result for Western blot analysis for Bax and BCL-2, caspase 3 and cleavage caspase 3. **b** Semiquantitative analysis of ten animals studied in each group. The relative amounts of Bax, BCL-2, caspase 3 and cleavage caspase 3 in each group of rats were normalized by β-actin. ***P < 0.001, **P < 0.01, *P < 0.05 (IRI vs. sham); ^#^ *P* < 0.05 (IRI vs. EGCG)

To investigate the effect of EGCG on apoptosis, we examined the expression of Bax, BCL-2, caspase 3 and cleavage caspase 3 in renal tissue. These results showed that compared with the sham group, the IRI group has higher levels of cleavage caspase 3 and Bax and lower levels of caspase 3 and BCL-2 (*P* < 0.05). However, EGCG treatment can significantly attenuate the tread as demonstrated by lower levels of cleavage caspase 3 and Bax and higher levels of caspase 3 and BCL-2 in the EGCG group than in the IRI group (*P* < 0.05). This indicated that EGCG treatment ameliorates I/R-induced apoptosis in renal ischemia–reperfusion injury (Fig. 5).

### EGCG pretreatment can suppress NF-κB activation in renal IRI

**Fig. 6:**
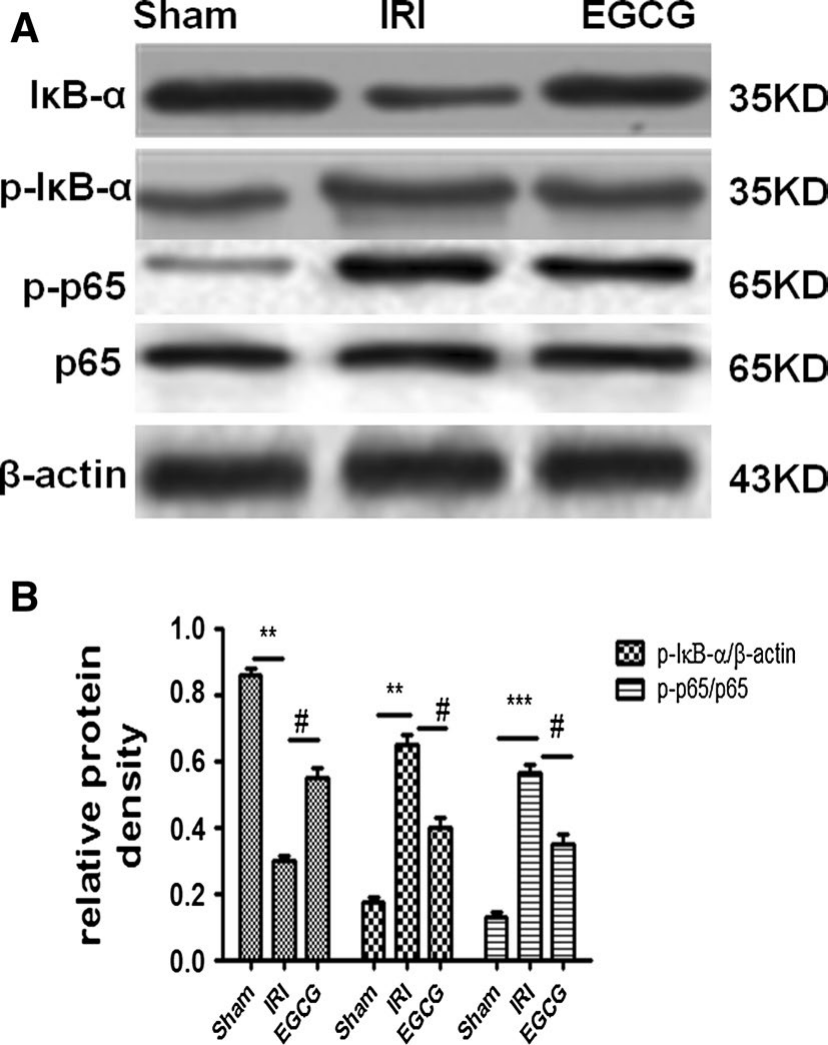
Effects of EGCG pretreatment on the NF-κΒ activation after renal IRI. Western blot analysis was employed to the expression of IκB-α and p65. **a** A representative result for Western blot analysis of IκB-α, p-IκB-α, p-p65 and p65. **b** Semiquantitative analysis of ten animals studied in each group. The relative amounts of IκB-α and p-IκB-α in each group of rats were normalized by β-actin; the relative amounts of p65 and p-p65 in each group of rats were normalized by β-actin and presented as a ratio of p-p65 to p65. ***P < 0.001, **P < 0.01 (IRI vs. sham); ^#^ *P* < 0.05 (EGCG vs. IRI)

To further study the mechanism of EGCG pretreatment decreasing inflammation and apoptosis, we assessed NF-κB activation by measuring IκB-α and p65. Compared with the sham group, the phosphorylation level of IκB-α and p65 and the degradation of IκB-α in the IRI group were significantly increased (*P* < 0.05). Therefore, EGCG can significantly attenuate the effect of I/R injury as displayed by lower phosphorylation levels of IκB-α and p65 (p-IκB-α and p-p65) and the lower degradation level of IκB-α in the EGCG group than in the IRI group (*P* < 0.05). Moreover, neither I/R nor EGCG treatment has influence on the expression of p65. These results indicated that EGCG pretreatment could attenuate NF-κB activation upon IRI (Fig. 6).

### EGCG pretreatment significantly promotes p38 activation in renal IRI

**Fig. 7:**
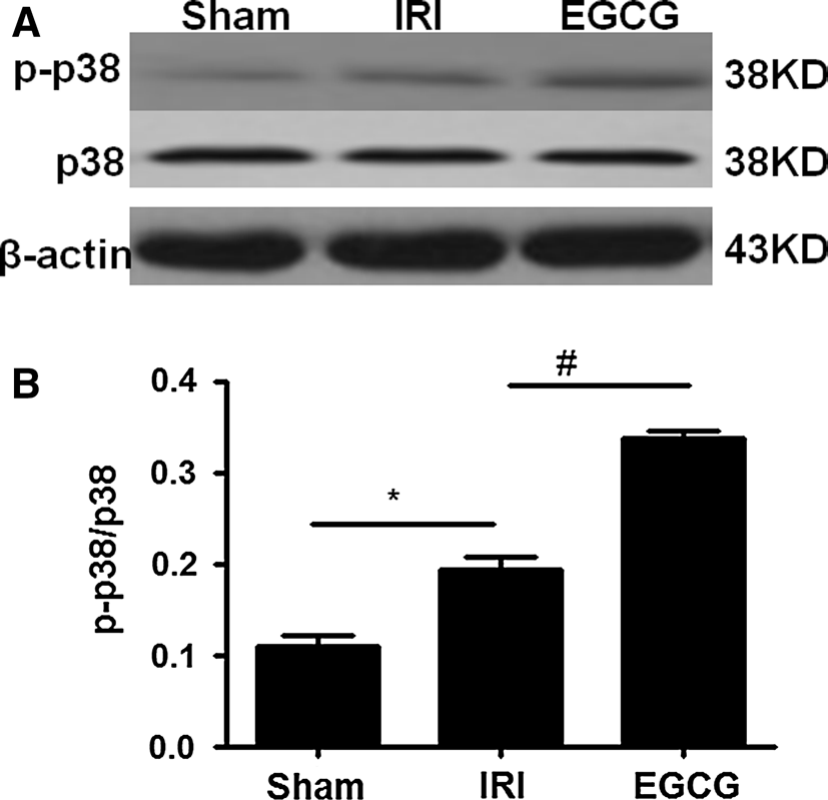
Effects of EGCG pretreatment on the p38 signaling after renal IRI. Western blot analysis was employed to the expression of p-p38 and p38. **a** A representative result for Western blot analysis of p38. **b** Semiquantitative analysis of 10 animals studied in each group. The relative amounts of p38 and p-p38 in each group of rats were normalized by β-actin and presented as a ratio of p-p38 to p38. *P < 0.05 (IRI vs. sham); ^#^ *P* < 0.05 (EGCG vs. IRI)

As the next step to explore the underlying mechanisms of EGCG decreasing NF-κB activation in IRI, we selectively examined the p38 signaling in renal I/R injury. These results showed that neither I/R nor EGCG treatment has influence on the expression of p38 (*P* < 0.05) (Fig. 7). But, compared with sham group, I/R injury can significantly induce p38 activation as demonstrated by higher levels of p-p38 in IRI group. Moreover, EGCG treatment can further promote p38 activation as shown by higher levels of p-p38 in EGCG group than in IRI group (*P* < 0.05) (Fig. 7). Our data indicated that EGCG pretreatment could induce p38 activation upon IRI.

## Discussion

Our study showed that EGCG can protect kidneys from renal I/R injury in a rat model. The renoprotective effect of EGCG seems to be associated with its potent anti-apoptotic and anti-inflammatory properties, which affect various pathophysiologic pathways inducing I/R injury.

Renal ischemia–reperfusion injury takes up a major role in the cause of AKI, which leads to high morbidity and mortality in patients inflicted to shock, renal transplantation or renal reaction [1]. It has been demonstrated that inflammation and apoptosis play vital roles in renal I/R injury [2] and therapeutic strategies targeting apoptosis and inflammation appear to reduce renal injury.

EGCG, a target of the research, has been demonstrated to have antioxidative, anti-apoptotic and anti-inflammatory properties [6, 7]. It comprises about 9–13 % content of the green tea. A recent research has shown that EGCG has protective effect in testicular torsion I/R injury by suppressing inflammation [11]. In this study, we demonstrated that EGCG treatment at the concentration of 50 mg/kg significantly decreased I/R-induced renal dysfunction and histopathologic change as demonstrated by low Cr and BUN levels, degeneration tubular architecture, tubular cell swelling, tubular dilation, tubular necrosis and inflammatory cell infiltration. This clearly demonstrated that EGCG treatment can decrease renal I/R injury, which is in line with a previous study [12], and if we persist in drinking green tea for a long time, levels of EGCG with renoprotective effect can be reached.

To explore the mechanism of EGCG decreasing renal I/R injury, we examined its influence on inflammation and apoptosis. The abnormal inflammation and apoptosis in renal I/R injury induce the production of massive pro-inflammatory cytokines and cell apoptosis, which in turn further exaggerated inflammation and cell apoptosis, leading to serious apoptosis and necrosis in the kidney [3]. In our study, we found that I/R injury can induce the increase in pro-inflammatory cytokines TNF-α, IL-1β and IL-6, pro-apoptotic protein Bax and cleavage caspase 3, and the decrease in anti-apoptotic BCL-2 and caspase 3. However, EGCG treatment can suppress the action of I/R injury as demonstrated by lower levels of TNF-α, IL-1β, IL-6, Bax and cleavage caspase 3 and higher levels of BCL-2 and caspase 3 in EGCG group than in IRI group. These results demonstrated that EGCG can attenuate renal dysfunction by suppressing apoptosis and inflammation.

Nuclear factor-κB (NF-κB) is an important nuclear transcription factor, which can play an important role in regulating inflammation and apoptosis [4, 5]. Moreover, its activation is dependent on p65 phosphorylation [4, 5]. In our study, we further demonstrated that renal I/R injury can induce IκΒ degradation and IκΒ and p65 phosphorylation. Therefore, EGCG can suppress the effect of I/R injury as shown by lower levels of p-IκΒ and p-p65 and higher levels of IκΒ in EGCG group than in IRI group. This indicated that EGCG can suppress NF-κB activation in renal ischemia–reperfusion injury.

The p38 MAPK pathway is a classical signaling pathway than can regulate NF-κB activation [4, 5], and EGCG can regulate p38 signaing pathway [13]. Our study showed that neither I/R nor EGCG has influence on the expression of p38. But I/R can induce p38 activation as shown by higher levels of p38 phosphorylation (p-p38) in IRI group than in sham group. Pretreatment with EGCG can further induce p38 activation as displayed higher levels of p-p38 in EGCG group than in IRI group. This study demonstrated that blocking p38 can aggregate renal ischemia–reperfusion injury [14]. Therefore, additional studies to confirm that suppressing PI3 K/Akt signaling enhances I/R-induced renal injury were not carried out, given the capacity of EGCG treatment in decreasing renal I/R injury. We cannot eliminate whether other signaing pathways, such as Akt signaling pathway, are involved in EGCG’s renoprotective effect in renal I/R injury. Further studies are necessary to address other signaling pathways involved in EGCG treatment decreasing renal I/R.

In summary, we demonstrated that precondition with EGCG can protect rats against renal I/R injury. This protective effect is associated with suppressing NF-κB and mediating inflammation and apoptosis by inducing p38 activation. Therefore, our study suggests that EGCG may be an effective practical strategy to decrease renal I/R injury. If we keep drinking green tea, it may be an effective way to be healthy.
